# A survey of manual vacuum aspiration’s experiences among the new medical graduates in Thailand

**DOI:** 10.1186/1742-4755-10-49

**Published:** 2013-09-11

**Authors:** Rapeepong Suphanchaimat, Nongluk Boonthai, Sasikan Tangthasana, Weerasak Putthasri, Viroj Tangcharoensathien, Kamheang Chaturachinda

**Affiliations:** 1 International Health Policy Programme, the Ministry of Public Health, Nonthaburi, Thailand; 2 Woman Health and Reproductive Rights Foundation of Thailand, Bangkok, Thailand; 3 Department of Obstetrics and Gynaecology, Faculty of Medicine, Siriraj Hospital, Mahidol University, Bangkok, Thailand; 4 Department of Obstetrics and Gynaecology, Faculty of Medicine, Ramathibodi Hospital, Mahidol University, Bangkok, Thailand

**Keywords:** Induced abortion, Vacuum curettage, Medical education, Thailand

## Abstract

**Background:**

Despite Thai laws permitting abortion conducted by registered medical practitioners, unsafe abortion still kills and maims Thai women as a result of inadequate access to safe abortion services. Surgical evacuation of the uterus by manual vacuum aspirator (MVA) is a safe and effective technique recommended by the World Health Organization (WHO) guidelines. This study assessed new medical graduates’ MVA experiences during their clinical years in medical schools.

**Methods:**

Cross-sectional questionnaire surveys on all new medical graduates participating in the annual assembly arranged by the Ministry of Public Health in 2010 and 2012 were applied. Descriptive and inferential statistics were employed for data analysis.

**Results:**

The significant minority of new graduates (44% and 43% in 2010 and 2012 batches) had seen but never used MVA. The proportion of graduates who had ‘never seen’ reduced from 32% in 2010 to 23% in 2012 while the proportion of ‘ever used’ had noticeably increased from 24% to 34% in corresponding years. Graduates from medical schools outside Bangkok and vicinity and those reporting confidence in their surgical skills tended to have more MVA experience. The 2012 graduation year was also positively related to higher experience on MVA.

**Conclusion:**

Though the proportion of graduates who had ever used MVA was still low in 2012, a positive change from that in 2010 was observed. Medical schools outside Bangkok and vicinity provided more opportunities for learning MVA. It is recommended that medical schools, especially in Bangkok and vicinity should provide more MVA learning opportunities for students. Adequate training and regular hands-on MVA practice should be incorporated into a wide range of clinical practice.

## Background

Maternal mortality is one of the key Millennium Development Goal (MDG) targets. Of several causes leading to maternal morbidity and death, unsafe abortion is undeniably the main contributor and considered as a global threat [1]. Although the global incidence saw a significant decreasing trend, from 35 abortions per 1,000 women aged 15-44 years in 1995 to 28 in 2008; the unsafe abortion rate was still high, at around 49% of all abortions in 2008, even higher than the 44% rate reported in 1995 [2]. Unsafe abortion accounts for 13% of maternal mortality in developing countries; and accounts for 99% of estimated global maternal death taking place in developing nations [3,4], hampering achievement of MDG5 in various developing countries.

Maternal health in Thailand has suffered from unsafe abortion related problems for a long time. A study by Warakamin et al in 1999, conducted in 787 government hospitals, found that 28.5% of 45,990 cases were admitted into hospital as a result of induced abortion. Among these induced-abortions, one third developed serious complications, and despite the advancement of health system development in Thailand [5], 54% of the women suffering from such complications had an abortion performed by unqualified healthcare providers [6].

The World Health Organization (WHO) defines unsafe abortion as a procedure for terminating an unwanted pregnancy either by persons lacking the necessary skills or in an environment lacking the minimal medical standards, or both [7,8]. In the past few decades, abortion procedures, both invasive and non-invasive methods, have developed in leaps and bounds in-line with the increasing number of trained healthcare staff with the aim to reduce the prevalence and complications of unsafe abortion. Among various techniques, the manual vacuum aspiration (MVA) has been proven to be a safe and effective treatment of choice, it should replace the outdated dilatation and curettage (D&C) [9].

“*Vacuum aspiration is the recommended technique of surgical abortion for pregnancies of up to 12 to 14 weeks of gestation. The procedure should not be routinely completed by sharp curettage. Dilatation and sharp curettage (D*&*C), if still practiced, should be replaced by vacuum aspiration”*[10].

During an MVA procedure, a 60-ml hand-held syringe with a self-locking plunger is applied to produce the vacuum used for aspirating the conception products. MVA can be provided under local anaesthesia in an out-patient setting, avoiding the need for an operating theatre and the risks of general anaesthesia [11]. Based on its effectiveness, between 87 and 100 percent [12-17], MVA is recommended as the treatment of choice for first-trimester abortion while D&C should only be used when MVA is not available [18,19]. Further, MVA is an appropriate choice where there is no skilled gynaecologist available, in particular in rural areas; it can be safely provided by trained personnel such as nurses or midwives, with physician backup if needed; as demonstrated in various country settings such as Bangladesh, China, Nepal, South Africa and Vietnam [20]. No significant difference in complication rates was reported between patients who had undergone MVA provided by physicians and physician assistants [13,21].

In Thailand, most new medical graduates are bound to the Ministry of Public Health (MOPH); the Office of the Permanent Secretary of the MOPH assigns them to serve mandatory rural service in almost 800 district, and 77 provincial, hospitals all over Thailand [22]. As frontline doctors, their knowledge and skill in providing safe abortion is indispensable and should be developed before leaving medical school. However, the application of MVA is not widely known and is less popular than D&C among practitioners, including gynaecologists. This contradicts the regulation recently endorsed by the Thai Medical Council in 2010 that MVA is the first line and standard treatment of almost all intra-uterine evacuation including abortion [23]. Besides, experience on MVA may vary resulting from a number of factors, such as, personal confidence in obstetric competence, location of medical school, opportunities of having hand-on training, etc. Recent studies by Wilson NW et al. in 2008 [24] and Lumsden MA et al. in 2010 [25] highlighted that traditional medical schools were facing difficulties in offering basic obstetric and gynaecologic skill to students due to the factual work overload at the schools. Darney BG et al also suggested that family medicine residents were not routinely trained to manage miscarriages using MVA, but have the potential to increase access to this procedure [26]. Nonetheless, there was no clear evidence from domestic studies describing the current experience of graduates on MVA and factors associated with this experience.

This study therefore aimed to assess whether or not new medical graduates had ever seen and used MVA during their clinical years in medical school, the association between their personal and educational attributes, their confidence in their obstetric and surgical skills, and their MVA experience.

## Methods

### Study design

Two cross–sectional surveys were conducted with all new medical graduates during the annual assembly to choose hospitals for their mandatory rural services on 2 April 2010 and 1 April 2012. The meetings were jointly arranged by the MOPH and the health professional associations. The MOPH also uses these occasions for the compulsory selection of the MOPH workplaces for new graduates from among the vacant posts available.

### Population and sample size

The target population was made up of all new medical graduates participating in the aforementioned meetings, 1,545 and 1,697 in 2010 and 2012 respectively. The questionnaire was distributed to all these new graduates along with their registration documents. The totals of 576 and 754 graduates returned the questionnaire, therefore, these corresponded to a response rate of 37.3% and 44.4%.

### Questionnaire design

An anonymous self--administered questionnaire survey was applied. The questionnaire consisted of three parts: (1) personal characteristics including age, gender, location of the medical school attended, the mode of admission to medical education: normal track of national entrance examination or the special track for rural students under the Collaborative Project to Increase Production of Rural Doctors (CPIRD)^a^; (2) self-assessed competency in obstetrics and surgical skill was measured by level of confidence using the Likert scale, ranging from one (least confident) to five (most confident) and (3) experience in the use of MVA during clinical years was measured by discrete answer of ‘never seen ’, ‘ever seen but never used’ and ‘ever used’. The questionnaire was tested for content validity and finalized. Completed questionnaires were anonymously dropped in a collecting box.

### Data analysis

Data were analysed by using STATA/SE version XI. The level of confidence on obstetric and surgical skills was regrouped into ‘less confident’ (Likert scale 1 to 3) and ‘confident’ (Likert scale 4 to 5). Descriptive statistics was presented in mean and percentage. Inferential statistics using Pearson’s Chi-square was applied to demonstrate association between personal attributes and experience on MVA in each batch^b^. Multivariable analysis, using multinomial logistic regression, was performed to adjust for all confounding factors including ‘graduation year’ (2012 VS 2010). ‘Never seen’ was set as base outcome compared to ‘ever seen but never used’ and ‘ever used’. Results of the multivariable analysis were presented in terms of relative risk ratios.

## Results

### Personal characteristics and level of confidence

The two batches of new medical graduates had an average age of 24 years old and had low levels of confidence in both obstetric and surgical skills. Two thirds of the samples graduated from medical schools in Bangkok and vicinity. Around 70% of the new graduates were admitted through the normal track, see Table 1.

**Table 1:** Profiles of new medical graduates and level of confidence in 2010 and 2012 batches

**Characteristic**	**2010 batch (N=576)**	**2012 batch (N=754)**
Age—yr		
• Mean	24.3	24.1
• Standard deviation	1.2	0.9
Sex—no. (%)		
• Female	362 (63.1)	452 (60.5)
• Male	212 (36.9)	295 (39.5)
Location of medical schools—no. (%)		
• Bangkok and vicinity	345 (65.8)	442 (61.5)
• Regional	179 (34.2)	277 (38.5)
Mode of admission—no. (%)		
• Normal track	442 (76.9)	520 (69.2)
• CPIRD	133 (23.1)	232 (30.8)
Confidence in obstetric competency—no. (%)		
• Less confident	386 (70.4)	509 (71.0)
• Confident	162 (29.6)	208 (29.0)
Confidence in surgical skills—no. (%)		
• Less confident	348 (63.5)	468 (65.3)
• Confident	200 (36.5)	249 (34.7)

Note: Missing data were not presented herewith as their numbers were comparatively small.

### Experience on the use of MVA

A quarter of graduates in 2010 and a third of graduates in 2012 had ever used MVA, though a substantial proportion of them (44% and 43% respectively) had never used it and had only seen their teaching staff or senior doctors use it; this proportion remained stable between the two batches.

**Figure 1:**
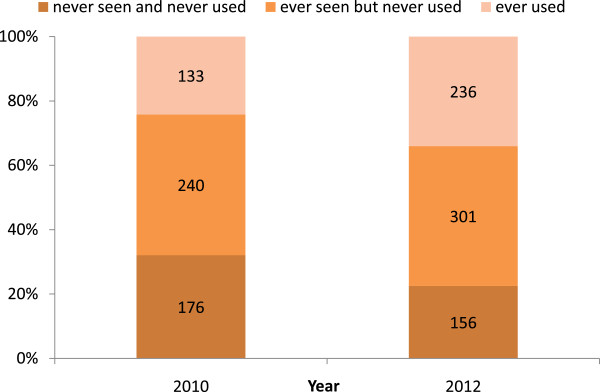
Experiences on MVA of new medical graduates in 2010 and 2012 batches.

Furthermore, 32% (176/549) of students in 2010 and 23% of (156/693) of students in 2012 had never seen MVA in their three clinical years in medical school. The proportion of ‘ever seen but never used’ did not change between the two batches. A positive change was observed, the proportion of graduates who had ‘never seen’ reduced from 32% to 23% while the proportion of ‘ever used’ had noticeably increased from 24% (133/549 students) in 2010 to 34% (236/693 students) in 2012; see Figure 1.

### Personal attributes and experience on MVA

The location of medical schools, modes of admission, and confidence in surgical skill were significantly related to MVA experiences in both 2010 and 2012 batches. In 2010, graduates from medical schools located outside Bangkok and vicinity had higher MVA experience in terms of having ‘ever used’ it, while around 87% of graduates who had ‘never seen’ MVA were from medical schools in Bangkok and vicinity. This finding was confirmed by the results from 2012 batch since around 70% of graduates who had ‘ever used’ MVA graduated from regional medical schools. The graduates admitted through the CPIRD programme tended to have higher MVA experience than those from the normal track. For surgical skill, the more they were confident in this competency, the more likely they were to have higher experience on MVA. However, this was not the case for obstetrics competency and MVA experience, see Table 2 and Table 3.

**Table 2:** Bivariate analysis showing the association between each personal attribute and the experience on MVA in the 2010 graduation year

**Characteristic**	**MVA experience**			**P-value**
	**Never seen**	**Ever seen &never used**	**Ever used**	
Sex—no. (%)				
• Female	106 (60.2)	158 (65.8)	84 (64.1)	0.497
• Male	70 (39.8)	82 (34.2)	47 (35.9)	
Location of medical schools—no. (%)				
• Bangkok and vicinity	132 (86.8)	136 (61.5)	60 (47.6)	<0.001**
• Regional	20 (12.2)	85 (38.5)	66 (52.4)	
Mode of admission—no. (%)				
• Normal track	152 (86.4)	181 (75.4)	87 (65.9)	<0.001**
• CPIRD	24 (13.6)	59 (24.6)	45 (34.1)	
Confidence in obstetric competency—no. (%)				
• Less confident	129 (74.6)	168 (70.9)	84 (63.6)	0.113
• Confident	44 (25.4)	69 (29.1)	48 (36.4)	
Confidence in surgical skills—no. (%)				
• Less confident	124 (71.7)	141 (59.5)	80 (60.6)	0.029*
• Confident	49 (28.3)	96 (40.5)	52 (39.4)	

Note: *Statistical significance over 95% level of confidence.

**Statistical significance over 99.9% level of confidence.

**Table 3:** Bivariate analysis showing the association between each personal attribute and the experience on MVA in the 2012 graduation year

**Characteristic**	**MVA experience**			**P-value**
	**Never seen**	**Ever seen &never used**	**Ever used**	
Sex—no. (%)				
• Female	97 (62.6)	174 (57.8)	147 (62.6)	0.447
• Male	58 (37.4)	127 (42.2)	88 (37.4)	
Location of medical schools—no. (%)				
• Bangkok and vicinity	138 (89.6)	206 (71.3)	70 (32.1)	<0.001**
• Regional	16 (10.4)	83 (28.7)	148 (67.9)	
Mode of admission—no. (%)				
• Normal track	125 (80.6)	23 (74.1)	138 (58.7)	<0.001**
• CPIRD	30 (19.4)	78 (25.9)	97 (41.3)	
Confidence in obstetric competency—no. (%)				
• Less confident	119 (72.3)	214 (71.3)	158 (67.2)	0.101
• Confident	35 (22.7)	86 (28.7)	77 (32.8)	
Confidence in surgical skills—no. (%)				
• Less confident	120 (77.9)	205 (68.3)	125 (53.2)	<0.001**
• Confident	34 (22.1)	95 (31.7)	110 (46.8)	

Note: *Statistical significance over 95% level of confidence.

**Statistical significance over 99.9% level of confidence.

Table 4 examines personal attributes in association with higher MVA experiences when controlled by all confounding factors. Clearly, three personal characteristics, i.e., (1) graduation from medical schools outside Bangkok and vicinity, (2) being confident in surgical skill, and (2) 2012 graduation year, were positively related to higher MVA experience with statistical significance, either considering ‘ever used’ or ‘ever seen but never used’ as outcome of interest . This association was prominent in ‘ever used’ as graduates from medical schools outside Bangkok and the vicinity had around 11 times more likely to have this experience compared to those from medical schools in Bangkok and vicinity. Confidence in surgical skill and the 2012 graduation year also contributed to about 1.3 to 1.8 times more chance of having exposed to MVA.

**Table 4:** Multivariable analysis determining the association between personal attributes as well as graduation year and the experience on MVA (‘Never seen’ as base outcome)

**Ever seen but never used**	**RRR**^**†**^	**P-value**	**95% CI**^**‡**^	
Male (VS female)	0.980	0.894	0.724	1.326
Medical school outside Bangkok (VS in Bangkok and vicinity)	3.698	<0.001***	2.430	5.628
CPIRD (VS normal track)	1.059	0.775	0.714	1.570
Confident in obstetrics (VS less confident)	1.055	0.777	0.728	1.529
Confident in surgical skill (VS less confident)	1.447	0.044*	1.010	2.073
2012 graduation year (VS 2010 graduation year)	1.345	0.048*	1.003	1.805
**Ever used**	**RRR**	**P-value**	**95% CI**	
Male (VS female)	0.854	0.385	0.598	1.220
Medical school outside Bangkok (VS in Bangkok and vicinity)	11.845	<0.001***	7.614	18.428
CPIRD (VS normal track)	1.122	0.602	0.727	1.732
Confident in obstetrics (VS less confident)	1.130	0.575	0.736	1.736
Confident in surgical skill (VS less confident)	1.744	0.008**	1.153	2.637
2012 graduation year (VS 2010 graduation year)	1.845	0.001**	1.303	2.612

Note: *Statistical significance over 95% level of confidence.

**Statistical significance over 99% level of confidence.

***Statistical significance over 99.9% level of confidence.

†*RRR* Relative risk ratio.

‡*95% CI* 95% Confidence interval.

## Discussions

The evidence clearly indicates that not all new medical graduates had ever experienced the use of MVA either by observation or hand-on training, despite the Thai Medical Council stipulating in 2010 that experience of MVA is a requirement for a medical license. Overall, factors significantly contributing to the higher MVA experiences were location of medical schools outside Bangkok and vicinity, CPIRD admission, and confidence in surgical skill. However, detailed analysis suggested that of medical schools outside Bangkok and vicinity, confidence in surgical skill, and the 2012 graduation year were factors significantly linked to higher MVA experience.

Our finding is similar to the findings from other international studies. For instance, Mhlanga in South Africa and Darney BG from the United States highlighted that where the MVA technique was a method of choice but few doctors were familiar with its application [26,27], despite the guideline recommended them to do so [28]. Darney BG also suggested that the inclusion of support staff in training and effective champions facilitated successful implementation of MVA services [26]. Relevant studies from international literatures also suggested that junior doctors who spent undergraduate years training at smaller/rural medical schools felt more confident and better prepared at internship since they had greater chances for more hands-on experience and more patient contact [29,30]. Thus medical schools with smaller size and less production capacity [31,32], most of which were established outside Bangkok and vicinity, might benefit from this point, since fewer residents undertaking their specialty training would mean the increased opportunities to handle case by medical students [33]. In addition, patients in the university hospitals in Bangkok and vicinity tend to present with more complicated problems, and a number of them are referred from elsewhere; they therefore commonly require specialist services, not easily handled by medical students [33].

Besides, the integration of MVA into the medical curriculum, especially by medical schools outside Bangkok, has come about as the result of the efforts made by various organizations concerning maternal and child health in recent years: the Royal Thai College of the Obstetricians and Gynaecologists, the Thai Health Promotion Foundation and the Woman Health and Reproductive Rights Foundation. Such efforts comprise a variety of activities, for instance, organizing annual national and international meetings in order to enhance recognition of threats to the health of women resulting from unsafe abortion, convening training workshops for healthcare providers to improve their knowledge, skill and attitudes in caring for abortion patients through state-of-the-art abortion technology, e.g. MVA and relevant treatments, working through the media, e.g. pamphlets, the internet and fact sheets with an objective to increase public concern. However, before expanding to the medical schools in Bangkok and vicinity, most activities in the inception phase were focused on regional medical schools where hand-on training for students can more easily and readily provided [23,33].

A few limitations were identified. Though cross sectional design is commonly used to infer causation, it cannot provide hard evidence on causal relationship compared to other stringent designs such as case-control or cohort [34]. Although the graduate survey has been performed annually, the MVA data was collected only in 2010 and 2012 survey. As a consequence, the two cross-sectional survey results cannot strongly determine a trend of MVA knowledge and experience in the target population. The authors recognised this important issues as rooms for improvement and had agreed to collect MVA data on an annual basis, as a regular monitoring on progress in this area. Results from the further upcoming annual surveys will provide more solid evidence on the trend of MVA knowledge and experience as well as other clinical competencies among new medical graduates.

Despite the fact that, the study encompassed a significant amount of the new medical graduate population, it was not possible to recruit students who opted out from the mandatory rural service scheme by paying a fine of approximately USD$14,000 [22] or by selecting their career path in health facilities not affiliated to the Office of the Permanent Secretary, the MOPH, e.g. military hospitals or psychiatric hospitals, as they did not show up; these groups accounted for approximately 20% of the total graduates [32,33].

Curriculum design and pedagogic learning methods in each medical school may vary significantly according to its contextual environment, and this can have a great impact on the competency of graduates. Interestingly, the self-reported confidence in obstetric competency and surgical skill among new graduates were relatively low, despite the fact that they were all qualified through the National License Examination. A thorough study determining the correlation between curricula design and the competency in reproductive healthcare among a variety of health professionals, taking into account the potential limitations mentioned above, is thus recommended. In summary, adequate training and regular hand-on practice is needed and should be incorporated into a wide range of practices, from a family practice to advanced gynaecologic care [35,36].

## Conclusion

Though the proportion of graduates who had ever used MVA was still low in 2012, a positive change from that in 2010 was observed. Factors significantly contributing to the higher MVA experiences were location of medical schools outside Bangkok and vicinity, confidence in surgical skill and graduation in 2012. Medical schools, especially in Bangkok and vicinity should provide more MVA learning opportunities for students. Adequate training and regular hands-on MVA practice should be incorporated into a wide range of clinical practice.

### Ethics approval

While informed consent was sought and protection of confidentiality was strictly followed; the National Ethical Review Committee waived ethical clearance as this is a regular monitoring work by the Government as shown in the letter Ref IHRP 47.2/2553 date 28 January 2553 BE (2010 AD).

## Endnotes

^a^Students under the CPIRD program spend one year on basic sciences, and two pre-clinical years in university, followed by three clinical years in the MOPH affiliated teaching institutes, mostly accredited regional and provincial hospitals where teaching associates were their clinical teachers. All students recruited through the normal track and CPIRD were subject to pass all three parts of the national licensing examination in years 3, 5 and 6 to become licensed physicians.

^b^‘Batch’ in this study refers to students graduating in that calendar year, not academic year. Therefore, the 2012 batch refers to students graduated in 2012 calendar year.

## Competing interests

The authors declare that they have no competing interests.

## Authors’ contributions

The study was designed by NB, WP, VT and KC. NB, RS, ST, and KC reviewed the literatures. Data collection and analysis were done by RS and WP. All authors contributed to drafting and revision and agreed upon the manuscript. All authors read and approved the final manuscript.
